# Improving Performance of an Integrated Solar Flow Battery by Cr- and Cu-Doped TiO_2_ Photoelectrodes

**DOI:** 10.3390/molecules28010171

**Published:** 2022-12-25

**Authors:** Zihan Zhang, Ping Lu, Tong Zhao, Huaneng Su, Qian Xu

**Affiliations:** Institute for Energy Research, Jiangsu University, Zhenjiang 212013, China

**Keywords:** Cu-TiO_2_, Cr-TiO_2_, photoelectrodes, integrated solar flow battery, ion doping

## Abstract

This work reports on the preparation of Cr-doped TiO_2_ (Cr–TiO_2_), Cu-doped (Cu-TiO_2_), and its utilization in the photoanode of a solar redox flow battery (SRFB). A pure TiO_2_ electrode, Cr-doped TiO_2_ electrode, and Cu-doped TiO_2_ electrode coated with different layers are prepared by the sol-gel method. XRD, XPS, and SEM are used to characterize the relevant data of the electrode. All three electrodes show the structure of the anatase phase, but the Cu-TiO_2_ and Cr-TiO_2_ electrodes are more crystalline. Using these materials as photoelectrodes to prepare integrated solar flow cells, the semi-cell and full-cell tests show that the doping of Cr and Cu improves the efficiency and charging current of solar cells. The average charging currents of the Cu-TiO_2_ and Cr-TiO_2_ electrodes are 384.20 μA and 450.75 μA, respectively, compared with the TiO_2_ electrode; this increment reaches values of 71.23% and 100.97%.

## 1. Introduction

At present, due to the emergence of environmental pollution, energy shortages and other crises, solar energy, wind energy and other clean energies are favored. Among them, solar energy has been widely studied because of its low cost and nearly unlimited reserves. The practical application of solar energy not only requires efficient energy conversion but also requires large-scale energy storage facilities with low construction costs to cope with the shortcomings of solar energy’s intermittent nature. Among the many solar energy application technologies, dye-sensitized solar cells (DSSC) have been widely studied due to their low cost, wide range of raw material sources and simple processes. Flow batteries have become a promising method for the efficient use of solar energy due to their low costs, strong safety, and individually adjustable output power and capacities. However, traditional solar flow batteries are complex in structure, and their conversion efficiency is low. Therefore, integrating the conversion and storage of solar energy into a single device has become an attractive approach. To this end, an integrated solar redox flow battery (SRFB) integrating solar energy with redox flow batteries was conceived [1,2,3,4,5,6,7]. The SRFB’s unique integrated design can directly convert solar energy into chemical energy for storage, eliminating the need for intermediate conversion into electrical energy. At the same time, the integrated design eliminates the need for maximum power point tracking equipment and direct current (DC) inverters for photovoltaic panels, thereby increasing efficiency while potentially reducing construction and maintenance costs.

Although the history of integrated solar flow batteries is short, SRFB’s performance and equipment research have evolved greatly. For example, many researchers have systematically studied and elaborated on the mechanisms and design methods of solar flow batteries [8,9,10]. Researchers have also used perovskite/silicon tandem solar cells to improve the performance of solar flow cells [11]. In addition to the above studies, the effects of thermo-electrochemical effects on the optical charging performance of SRFBs have also come to researchers’ attention [12]. Some researchers have used 4-hydroxy-2,2,6,6-tetramethylpiperidine-1-oxy (4-OH-TEMPO) [7] and methyl violet (MV) as the anode and cathode, respectively, of an integrated SFB device and III-V materials [13] as the photoelectrode for the battery. However, the material of this photoelectrode is extremely susceptible to photo corrosion, which greatly limits the service life of the integrated SRFB. 

In addition to the above-mentioned ways to improve the performance of SRFB by changing redox couples, the improvement of photoelectrodes has gradually attracted the attention of researchers [14,15,16]. As one of the widely used photocatalytic materials, TiO_2_ has the characteristics of chemical stability, abundant natural reserves and low cost. However, its wide bandgap (3.3 eV) results in its lower solar utilization in SRFB. To address this issue, many studies use cryogenic hydrolysis [17], microwave-assisted hydrothermal [18] or sol-gel [19,20,21,22,23,24]-doped Cu^2+^ on the surface of the TiO_2_ electrode. In addition to Cu^2+^, Cr^3+^ [25,26,27,28,29] is also widely used to improve the performance of the battery. Chen [30] et al. used Ga as a doped element to surface modify the TiO_2_ photoelectrode. The results of Liu [31] et al. showed that the doping of Ta element can obtain a higher solar transcoding efficiency than Ga element doping. In addition to this, Tian [32] et al. modified the photoelectrode using MoS_2_ and eventually achieved a solar-to-output energy conversion efficiency (SOEE) of 0.17% and 4.78% for SRFB at electrolyte concentrations of 0.1 M and 1 M. Among these methods, metal ion doping was found to be effective in reducing the TiO_2_ band gap and had a certain stability, making it attractive for improving solar energy utilization efficiency. Therefore, the application of metal ion-doped TiO_2_ photoelectrodes to SRFB is a successful attempt to improve battery performance and solar energy utilization. 

The above example clearly shows that the ion doping of the photoelectrode can significantly improve its electrochemical properties. In this work, inspired by the above research, the advantages and disadvantages of different doping methods are fully considered. A solar redox flow battery with a Cu^2+^- and Cr^3+^-doped TiO_2_ photoelectrode was proposed for solar energy storage and used FeCl_2_ and CrCl_3_ as redox couples. Scanning electron microscopy (SEM), X-ray diffractometry (XRD), and X-ray photoelectron spectroscopy (XPS) characterized the physicochemical properties of the sample. At the same time, the electrochemical workstation was used to evaluate the performance of the developed SRFB under different parameter conditions. 

## 2. Results and Discussion

### 2.1. Half−Cell Analysis

**Figure 1:**
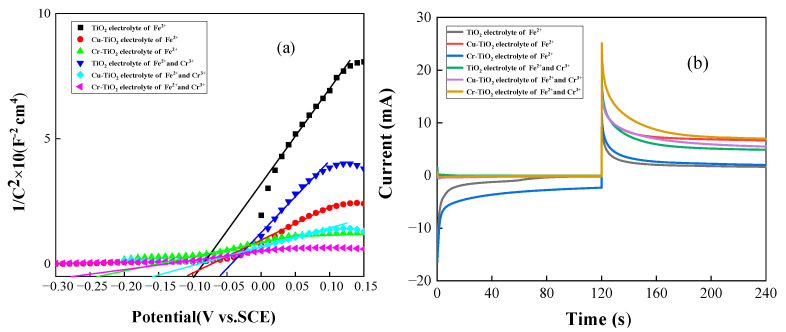
(**a**) The Mott-Schottky curve of metal ion-doped TiO_2_ electrode in different electrolytes; (**b**) instantaneous current plot of metal ion-doped TiO_2_ electrode in different electrolytes.

Figure 1 shows the Mott–Schottky curve and transient current curve of the TiO_2_ photoelectrode doped with Cu^2+^ and Cr^3+^ in different electrolytes. In Figure 1b, the transient current curve with no applied bias is within 0–120 s, and the transient current curve with an applied 0.5 V bias is within 120–240 s. The specific data in Figure 1b are listed in Table 1 and Table 2. Based on the Mott–Schottky curve and relation, the carrier concentration and flat-band potential of the three photoelectrodes were calculated, where $N_{D}$ is the carrier concentration, $e_{0}$ is the fundamental charge, $\varepsilon_{TiO_{2}}$ is the relative dielectric constant of TiO_2_, $\varepsilon_{0}$ is the dielectric constant, and m is the slope of the curve.

(1) $$N_{D}=-\frac{2}{e_{0}\varepsilon_{TiO_{2}}\varepsilon_{0}m}$$

Among them, the flat band potential *E_fb_* equaled -0.1 V for the TiO_2_ photoelectrode in the Fe^2+^ electrolyte, and the flat band potential *E_fb_* was -0.057 V in the Fe^2+^-Cr^3+^ mixed electrolyte. The Cu-TiO_2_ photoelectrode in the Fe^2+^ electrolyte had a flat band potential *E_fb_* of -0.11 V, while in the Fe^2+^-Cr^3+^ mixed electrolyte, *E_fb_* = -0.16 V. The flat band potential *E_fb_* was -0.24 V for the Cr-TiO_2_ photoelectrode in Fe^2+^ electrolyte, and the flat band potential *E_fb_* was -0.277 V in the Fe^2+^-Cr^3+^ mixed electrolyte. According to the relational formula (1) and Figure 1a, it can be seen that the carrier concentration of the TiO_2_ photoelectrode doped with metal ions is significantly higher than that of the TiO_2_ photoelectrode. At the same time, the Cr-TiO_2_ photoelectrode has the largest carrier concentration *N_D_*=1.5 × 10^21^ cm^−2^ in the Fe^2+^-Cr^3+^ mixed solution. Furthermore, according to Figure 1b, under the condition of the applied bias of 0.5 V, in the Fe^2+^-Cr^3+^ mixed solution, the average current of doped Cr^3+^ (8.93 mA) is about 43.8% higher than that of TiO_2_ electrodes (6.21 mA). This means that due to the doping of metal ions, the carrier concentration of the TiO_2_ photoelectrode becomes higher, and the oxidation capacity becomes stronger. In addition, the increase in the concentration of the carrier will also improve the conductivity of the photoelectrode, reduce the recombination rate of the photogenerated carrier, and thus increase the density of the photocurrent. This explains why the TiO_2_ photoelectrode doped with metal ions in Figure 1b has a higher current density. From the perspective of the flat band potential, the TiO_2_ photoelectrode doped with metal ions has a more negative flat band potential than the TiO_2_ photoelectrode. This can make the photoelectrode generate a higher open circuit voltage, which can improve the performance of the solar flow battery to a certain extent. 

**Figure 2:**
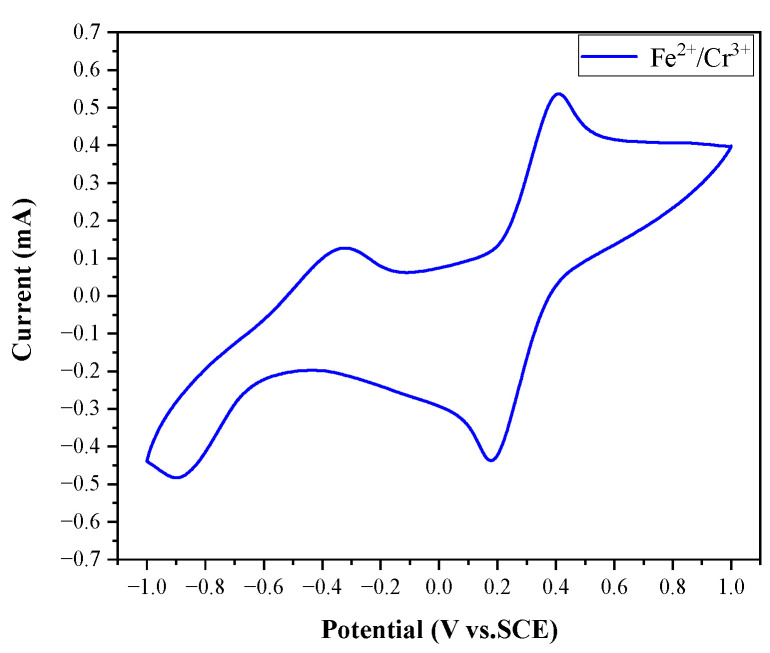
CV plot of Fe^2+^-Cr^3+^ electrolyte.

In addition to the above tests, we measured the cyclic voltammetry (CV) plot of the Fe^2+^ -Cr^3+^ electrolyte with a glass-carbon electrode as the working electrode, a Pt electrode as the counter electrode, and a saturated calomel electrode (SCE) as the reference electrode. The sweep rate was 10 mV/s. The CV plot is shown in Figure 2.

The redox processes of Fe^2+^/Fe^3+^ and Cr^2+^/Cr^3+^ redox pairs in the SRFB are summarized in Equations (2)–(4) as follows:

Anolyte:

(2) $$Fe^{2+}\underset{}{⇌}Fe^{3+}+e^{-}\;E_{0}=0.29\;V\;vs.\;SCE$$

Catholyte:

(3) $$Cr^{2+}\underset{}{⇌}Cr^{3+}+e^{-}\;E_{0}=-0.60\;V\;vs.\;SCE$$

Overall:

(4) $$Fe^{2+}+Cr^{3+}\underset{}{⇌}Fe^{3+}+Cr^{2+}\;E_{0}=0.89\;V\;vs.\;SCE$$

**Figure 3:**
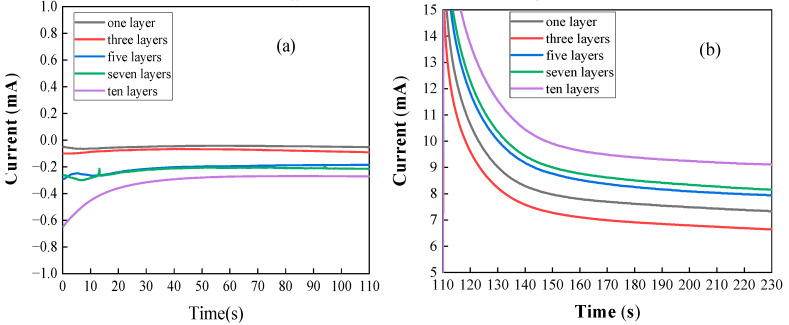
(**a**) I-t curve of Cu-TiO_2_ photoelectrode in the Fe^2+^ and Cr^3+^ electrolytes under 0 V bias; (**b**) I-t curve of Cu-TiO_2_ photoelectrode in the Fe^2+^ and Cr^3+^ electrolytes under 0.5 V bias.

According to the above analysis, the performance of TiO_2_ photoelectrodes doped with metal ions is significantly improved compared to the TiO_2_ photoelectrode. Therefore, in order to explore the effect of film thickness coated on the surface of the TiO_2_ photoelectrode on the performance, electrochemical measurements were made on the Cu-TiO_2_ photoelectrode coated with 1 layer, 3 layers, 5 layers, 7 layers and 10 layers, respectively. Figure 3 shows the I-t curve of the Cu-TiO_2_ photoelectrode at different biases in the Fe^2+^-Cr^3+^ electrolyte. As can be seen from the figure, in the Fe^2+^-Cr^3+^ electrolyte, without applying an external bias voltage, the 1-layer coating has the best charging performance, and the 10-layer coating has the worst performance. However, after applying a 0.5 V bias, the 10-layer TiO_2_ photoanode has the highest photocurrent curve at both photoexcitation and stabilization. At the same time, except for 1 layer and 3 layers, which are susceptible to the applied voltage, the remaining layers show a trend of better performance with more layers. This shows that in the current number of coatings, more coatings can allow TiO_2_ to produce more photoelectrons under the same light conditions, resulting in a higher charging current. In addition to the above reasons, the difference in the number of coating layers of the photoelectrode also affects the uniformity of metal ions on the surface of the photoelectrode and the transmittance of light. As the number of layers increases, the distribution of metal ions becomes more uniform, but this also leads to a decrease in light transmittance, which affects its performance. Therefore, a good balance needs to be found.

### 2.2. Full−Cell Testing

**Figure 4:**
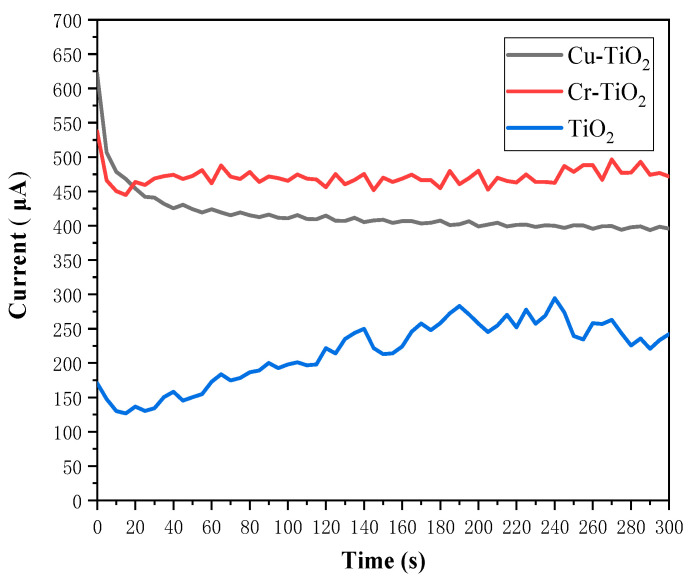
Charging currents of Cu-TiO_2_ photoelectrodes, Cr-TiO_2_ photoelectrodes and TiO_2_ photoelectrodes in the Fe^2+^ and Cr^3+^ electrolytes.

Figure 4 shows the Fe^2+^ and Cr^3+^ electrolyte (0.1 mol·L^−1^) Cu-TiO_2_ photoelectrode, Cr-TiO_2_ photoelectrode, and the TiO_2_ charging current of the photoelectrode at operating temperature (35 °C). Based on the curve in the figure, the average charging current of these two photoelectrodes under stable conditions is calculated. Among them, the average charging current of the Cu-TiO_2_ photodetector is 384.20 μA. The average charging current of the Cr-TiO_2_ photoelectrode is 450.75 μA. The average charging current of the TiO_2_ photoelectrode is 224.28 μA. The electrochemical test results show that with the doping of metal ions, the charging performance of photochemical batteries has been significantly improved. Among them, the current of Cu-TiO_2_ photoelectrode was increased by 71.23%, and the Cr-TiO_2_ photoelectrode was increased by 100.97%. This is in line with the results of the Mott–Schottky test in the previous section. That is, the carrier concentrations of the Cu-TiO_2_ and Cr-TiO_2_ photoelectrodes are significantly higher than that of the TiO_2_ electrode, while the two have a more negative flat band potential, allowing them to obtain higher open-circuit voltages and electron injection driving forces. In addition, the improvement in charging performance may also be due to the addition of Cu^2+^ and Cr^3+^, so that the recombination rate of photogenerated carriers decreases during charge transfer.

Based on the test results of the full battery, we calculated the average SOEE for this battery by using Equation (5) [33]:

(5) $$SOEE\;(\%)=(\int I_{dis}\times dt\times \Delta E_{0})/(P_{in}\times S\times t)\times 100$$

where $I_{dis}$ is the discharge current density, $\Delta E_{0}$ is the potential difference of the two redox couples in the reversible state, t is the illumination time, $P_{in}$ is the incident solar power, and S is the area of the solar flow cell window.

According to Equation (5), the average SOEE of the Cr-TiO_2_ photoelectrode at 0.1 M Fe^2+^-Cr^3+^ electrolyte is 0.501%, higher than the MoS_2_-modified photoelectrode [32] at the same electrolyte concentration. 

### 2.3. XRD Analysis

**Figure 5:**
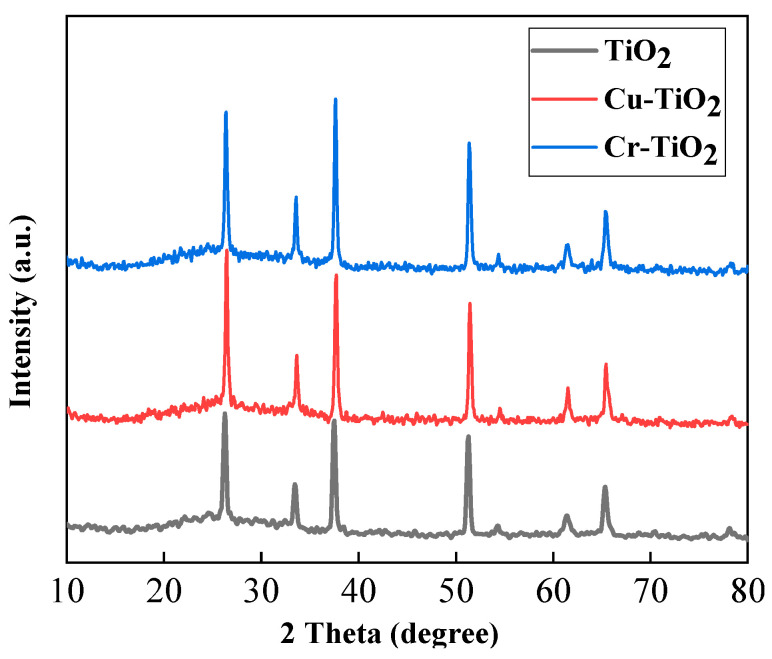
XRD pattern of TiO_2_ photoelectrode, Cu-TiO_2_ photoelectrode, and Cr-TiO_2_ photoelectrode.

Figure 5 shows the XRD diagram of the prepared TiO_2_, Cr-doped TiO_2_ and Cu-doped TiO_2_ photoelectrodes. Regarding the three plots in the figure, comparing the three spectra in the figure, we can see that the diffraction peaks of the Cr-doped TiO_2_ and Cu-doped TiO_2_ photoelectrodes are basically the same as the diffraction peak of the TiO_2_ photoelectrode. However, it can be found that the peak shape of the Cr-doped TiO_2_ and Cu-doped TiO_2_ photoelectrodes is sharper and stronger than the peak shape of the TiO_2_ photoelectrode. This means that the crystallization quality of the film is better than that of the TiO_2_ photoelectrode at this time. This is because after doping Cu^2+^, the ion radius of Cu^2+^ is 0.072 nm, which is similar to Ti^4+^ (0.068 nm), so the doped metal ions can replace the lattice ions, and the lattice distortion is not obvious. From this, we can infer that CuO is formed on the surface of the photoelectrode at low temperatures, but because there is less doped Cu^2+^, it is not enough to form grains, so it is dispersed in TiO_2_ in the form of microparticles. Between the two particles, a Ti-O-Cu bond is formed. The formation of this chemical bond promotes the formation of the TiO_2_ anatase phase, thereby making the absorption peak shape of the detected (101) crystal face more acute, improving the crystallinity of the sample so that the crystallization tends to be intact.

### 2.4. XPS Analysis

**Figure 6:**
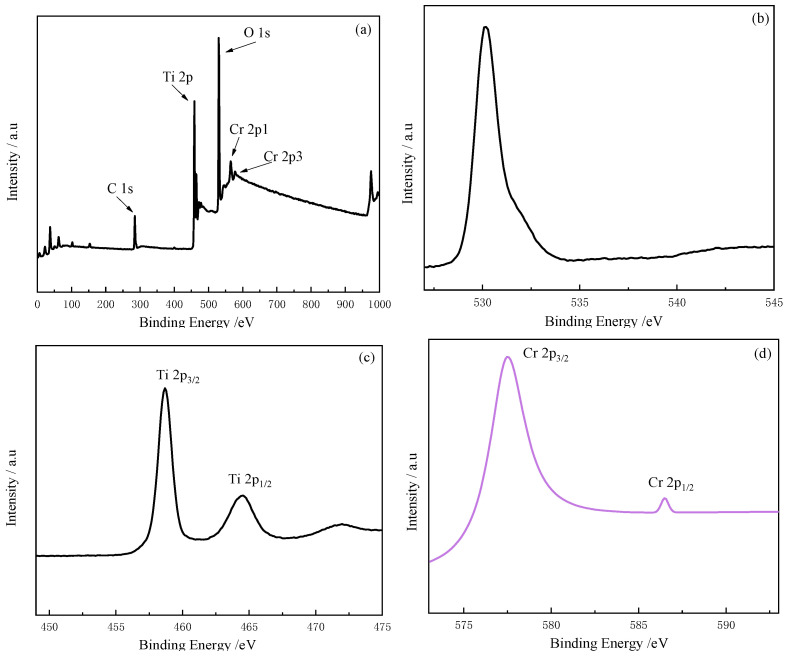
XPS spectra: survey for (**a**) Cr-TiO_2_; (**b**) O 1s; (**c**) Ti 2p; and (**d**) Cr 2p.

The O 1s peak was located at 530.18 eV with an asymmetric pattern, as presented in Figure 6b. The primary peak at 530.18 eV indicates the O^2-^, while the additional shoulder at a higher energy side implies surface OH^-^ groups or chemisorbed H_2_O. In the case of Ti 2p, two peaks were detected at 458.9 and 464.5 eV (Figure 6c), which correspond to Ti 2p_3/2_ and Ti 2p_1/2_, respectively. The binding energies were shifted toward the higher energy side compared to those of pure TiO_2_ (458.4 and 464.2 eV for Ti 2p_3/2_ and Ti 2p_1/2_, respectively). Thus, we assumed that Cr doping affected the chemical states of TiO_2_ by means of Cr substitution for Ti. The peaks at 577.5 eV and 587.0 eV (Figure 6d) were identified as Cr 2p_3/2_ and Cr 2p_1/2_, respectively, indicating the presence of Cr in the sample. Since oxidized chromium was not observed (as shown in Figure 6a), it was certain that Cr had been successfully implanted into the TiO_2_ lattice during the doping process. Therefore, we believe that Cr^3+^ were incorporated into the TiO_2_ lattice. 

## 3. Materials and Methods

### 3.1. Chemicals

Tetrabutyl titanate (Ti(C_4_H_9_O)_4_) (Shanghai Lingfeng Chemical Reagent Co., Ltd., Shanghai, China), acetylacetone (CH_3_COCH_2_COCH_3_) (Jiangtian Chemical Technology Co., Ltd., Tianjin, China), absolute ethanol (CH_3_CH_2_OH) (Sinopharm Chemical Reagent Co., Ltd., Shanghai, China), cupric chloride dihydrate (CuCl_2_·2H_2_O) (Sinopharm Chemical Reagent Co., Ltd., Shanghai, China) and polyethylene glycol (HO(CH_2_CH_2_O)_n_H) (Sinopharm Chemical Reagent Co., Ltd., Shanghai, China), hydrochloric acid (HCl) (Shanghai Aladdin Biochemical Technology Co., Ltd., Shanghai, China), ferrous chloride tetrahydrate (FeCl_2_·4H_2_O) (Sinopharm Chemical Reagent Co., Ltd., Shanghai, China), chromium trichloride hexahydrate (CrCl_3_·6H_2_O) (Sinopharm Chemical Reagent Co., Ltd., Shanghai, China) deionized water (H_2_O), and FTO Glass. All chemicals were analytical grade and used without further purification.

### 3.2. Preparation of TiO_2_ Sol, TiO_2_ Sol−Doped Cu^2+^ and TiO_2_ Sol−Doped Cr^3+^

The anhydrous ethanol and deionized water were mixed in a 5:1 ratio and stirred at a uniform speed of 30 min using a magnetic stirrer to form a homogeneous solution, which was named solution A. Furthermore, the tetrabutyl titanate solute, anhydrous ethanol solution and acetylacetone were mixed, stirred for 30 min using a magnetic stirrer until a homogeneous solution was formed, and named solution B. We mixed solution A with solution B, let it stand for a few minutes, and added concentrated hydrochloric acid (HCl) drop by drop; it was then stirred for 10 min in a magnetic stirrer. Polyethylene glycol was added to the mixed solution and placed in a magnetic stirrer for 30 min. The resulting mixed solution was sealed and aged at room temperature for 24 h for use. 

Cu-doped TiO_2_ sol was prepared using the same method. The difference was that different concentrations of the atomic solutes (CuCl_2_·2H_2_O) were added to solution A. 

### 3.3. Preparation of Electrodes

We used the following steps to prepare the photoelectrodes. First, the treated TiO_2_ sol and the TiO_2_ sol doped with Cu^2+^ were coated with a homogenizer, FTO glass (1 × 1.2 cm^2^). The actual coating area is controlled at 1 × 1 cm^2^. We placed the FTO glass in a drying box for 10 min (70 °C) for each coat for a total of seven coatings. Then, we put the coated FTO glass into a muffle furnace (400 °C) to isolate the oxygen from calcination for 1 h. Finally, the prepared photoelectrode was sealed and stored away from light.

**Figure 7:**
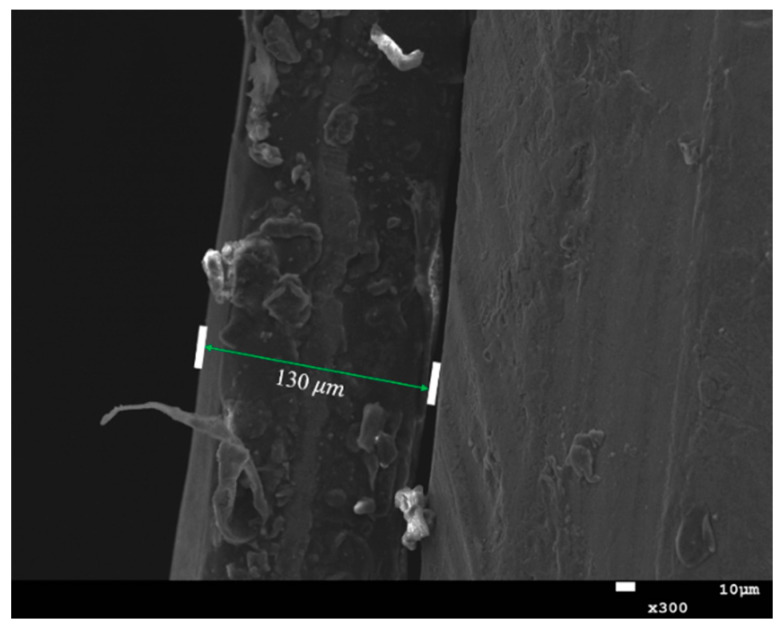
Cross-sectional view of the thin film on the surface of the Cu-TiO_2_ optical electrode.

Figure 7 shows the cross-section of the Cu-TiO_2_ photoelectrode surface film used in full-cell testing. According to the SEM diagram, it can be calculated that the thickness of the 7 layers coated by the photoelectrode was about 130 μm. 

### 3.4. Half−Cell and Full−Cell Testing

The experiment used the IVIUM electrochemical workstation for full-cell and half-cell testing. In the half-cell test, the Mott–Schottky curve, linear sweep curve and instantaneous current curve on the anode side of the SRFB were mainly measured. During the instantaneous current curve measurement, the current under five voltage gradients between 0 and 0.8 V was measured at 0.2 V intervals. Again, during full battery testing, an electrochemical station was used to measure the charging current of SRFB without external bias. The test time was 300 s, and multiple sets of experiments were repeated. 

In the testing of the full cell and half-cell, since this SRFB used a 1 cm^2^ size photoelectrode, therefore, the magnitude of the current measured during the experiment is the current density on the photoelectrode per unit area.

In addition, to avoid the effect of one side of the electrolyte penetrating the proton exchange membrane during the whole battery experiment and contaminating the electrolyte on the other side, resulting in degraded battery performance, an Fe^2+^-Cr^3+^ mixed electrolyte was used during the experiment so that even if one electrolyte penetrated through the membrane, the other electrolyte would not be contaminated. 

## 4. Conclusions

In this work, TiO_2_, Cu-doped TiO_2_, and Cr-doped TiO_2_ photoanodes were successfully prepared on FTO glass using the sol-gel method. Through the analysis of the battery performance test and the analysis of the characterization results, the following conclusions can be drawn:

(1) The charging currents of Cu-TiO_2_ and Cr-TiO_2_ electrodes were, respectively, increased from 224.28 μA to 384.21 μA and 450.75 μA**,** with an increase of 71.23% and 100.97%.
(2) The performance of the Cr-TiO_2_ electrode is significantly better than that of the Cu- TiO_2_ electrode and the TiO_2_ electrode. Its superior performance is not only reflected in the charging current but also in the carrier concentration and flat-band potential.
(3) In the XRD spectrum, at the diffraction peak (2θ = 25°), the doping of metal ions makes the absorption peak shape on the (101) crystal plane sharper, which promotes the formation of anatase-phase TiO_2_ and improves the crystallinity of the sample. This improves the carrier concentration of the photoelectrode and further reduces the recombination rate of photogenerated carriers. This explains why the electrochemical properties of the Cr-TiO_2_ electrode are better than those of the TiO_2_ electrode.

## Figures and Tables

**Table 1:** Instantaneous current plot of metal ion-doped TiO_2_ electrode in different electrolytes (0–120 s).

**Electrode**	**Electrolytes**	**Current Density (mA)**
TiO_2_	Fe^2+^	−1.1893
TiO_2_ Cu-TiO_2_ Cu-TiO_2_ Cr-TiO_2_ Cr-TiO_2_	Fe^2+^-Cr^3+^ Fe^2+^ Fe^2+^-Cr^3+^ Fe^2+^ Fe^2+^-Cr^3+^	−0.0052 −0.0788 −0.2635 −3.6027 −0.1196

**Table 2:** Instantaneous current plot of metal ion-doped TiO_2_ electrode in different electrolytes (120–240 s).

**Electrode**	**Electrolytes**	**Current Density (mA)**
TiO_2_	Fe^2+^	2.1843
TiO_2_ Cu-TiO_2_ Cu-TiO_2_ Cr-TiO_2_ Cr-TiO_2_	Fe^2+^-Cr^3+^ Fe^2+^ Fe^2+^-Cr^3+^ Fe^2+^ Fe^2+^-Cr^3+^	7.5290 2.7923 6.2000 6.9306 8.9176

## Data Availability

Not applicable

## Sample Availability

Not available.
